# Grafting enhances drought stress tolerance by regulating the proteome and targeted gene regulatory networks in tomato

**DOI:** 10.3389/fpls.2025.1591437

**Published:** 2025-08-20

**Authors:** Pritam Paramguru Mahapatra, Dong Won Bae, Michitaka Notaguchi, Sowbiya Muneer

**Affiliations:** ^1^ Horticulture and Molecular Physiology Lab, Department of Horticulture and Food Science, School of Agricultural Innovations and Advanced Learning, Vellore Institute of Technology, Vellore, Tamil Nadu, India; ^2^ School of Biosciences and Technology, Vellore Institute of Technology, Vellore, Tamil Nadu, India; ^3^ Central Instrument Facility, Gyeongsang National University, Jinju, Republic of Korea; ^4^ Department of Botany, Graduate School of Science, Kyoto University, Kyoto, Japan

**Keywords:** drought, gene-regulatory network, proteome, resistant, stress tolerance, tomato

## Abstract

Tomato (*Solanum lycopersicum*), a widely cultivated yet perishable crop, depends heavily on adequate sunlight and water for optimal growth and productivity. However, due to unavoidable environmental and climatic changes—particularly drought—its productivity has declined in recent years. Grafting, an ancient horticultural practice, is known to enhance yield and combat abiotic stress by regulating physiological and cellular processes. The present study investigated drought tolerance in tomato at both the proteomic and transcriptomic levels. During the initial physiological screening stage, two drought-resistant genotypes of *Solanum lycopersicum* were selected as rootstocks and drought-susceptible genotypes as scions. Among six genotypes evaluated under drought stress (based on relative water content, chlorophyll fluorescence, and stomatal conductance), graft combinations G1 and G4 demonstrated superior performance. These combinations were subsequently selected for molecular analyses to investigate gene expression patterns and stress-responsive pathways. Our findings revealed that grafting susceptible tomato genotypes onto resistant rootstocks mitigated the deleterious effects of drought stress by improving photosynthetic pigment levels and reducing oxidative stress. A proteomic investigation observed that grafting improved cellular responses, metabolic processes, and stress response pathways. Furthermore, transcriptomic studies of stress-related genes, including *DREB*, *WRKY*, *PIPs*, *SOD*, *CAT*, *APX*, *HSPs*, and *LOX*, revealed enhanced stress tolerance in the G1 and G4 graft combinations.

## Introduction

Water comprises up to 70%–90% of the fresh weight of plants and is necessary for their growth and development. Drought stress is expected to increase due to climate change, as dry and semi-arid regions now make up 40% of the world’s land area and are projected to exceed 50% by the end of the twenty-first century (Huang et al., 2016). Drought stress represents one of the most pervasive and detrimental abiotic factors limiting crop productivity and quality globally, posing a major threat to sustainable agriculture (Coşkun, 2023). Some cultivars with high yield potential under controlled conditions are unable to achieve this in the field due to inappropriate growing methods and stress conditions, despite ongoing efforts to develop high-yielding variants of numerous crops. Drought remains one of the main issues restricting agricultural productivity (Bahadur et al., 2023; Razi et al., 2024; Tomato Genome Consortium, 2012), particularly in arid and semi-arid areas where frequent drought and water scarcity are prevalent (Rouphael et al., 2012). Crop decline and reduced quality result from drought and water scarcity, whereas optimal moisture balance remains the most important breeding requirement for yield and quality (Bukhari et al., 2022; Diatta et al., 2021).

Drought inhibits cell differentiation and organ growth, which in turn reduces the rate of germination. These factors cause plants to grow slowly and produce less biomass (Iqbal et al., 2020). Under such conditions, the growth period sees a sharp decline in leaf expansion, shoot growth, and fresh shoot weight (Shin et al., 2021). Many plants under drought stress undergo morphological and anatomical changes in their roots, shoots, and leaves, with an increased root/stem ratio (Piazza et al., 2021; Habibi et al., 2022). Drought stress has detrimental impacts on the transcriptome (Razi and Muneer, 2023), proteome (Razi et al., 2021), and photosynthetic mechanisms (Mukarram et al., 2021; Mukarram et al., 2023).

Grafting is a widely used horticultural technique that joins the shoot of one plant (scion) to the root system of another (rootstock), often improving stress resilience. It is often utilized in commercial fruit and vegetable agriculture, landscaping, and studies on plant molecular movement (Fullana-Pericàs et al., 2018; Li S. et al., 2022). Grafting can enhance plant growth and development, maintain parental properties, alter branch structure, increase yield, improve fruit flavor and nutritional value, and enhance abiotic stress resistance (Sánchez-Rodríguez et al., 2013; Melnyk and Meyerowitz, 2015; Thomas and Frank, 2019; Ellenberger et al., 2021).

However, graft compatibility and survival rate remain significant challenges due to limited technical knowledge and the suitability of rootstocks and scions. Graft compatibility refers to the successful connection of vascular and non-vascular tissues at the graft interface. Studies on the physiological and molecular mechanisms of graft junctions have recently become a focus, including key responsive genes at healing sites (Melnyk et al., 2018; Notaguchi et al., 2020; Kurotani and Notaguchi, 2021; Thomas et al., 2022; Chambaud et al., 2023) and genetic information exchange between rootstocks and scions (Thomas and Frank, 2019; Yang et al., 2022; Cerruti et al., 2021; Okamoto et al., 2022).

Although the scion also affects the grafted plant (Han et al., 2013, 2019; Chen et al., 2020; López-Hinojosa et al., 2021), grafting increases drought resistance (Ellialtioglu et al., 2019; López-Serrano et al., 2019; Chen et al., 2020), which is largely determined by the rootstock. Although grafting and drought resistance have been explored in several studies, few have comprehensively addressed the relationship between the physiology and phenotype of grafted plants under drought stress and their molecular responses (Notaguchi et al., 2020; Kurotani and Notaguchi, 2021; Thomas et al., 2022; Chambaud et al., 2023; Suresh and Muneer, 2025).

The present study provides an overview of how grafting enhances drought stress tolerance in tomato by mobilizing micromolecules and proteins. It also enumerates the targeted genes and mobile molecules that respond to dryness and induce drought resistance in grafted plants.

## Materials and methods

### Plant material and treatments

In this experiment, four genotypes of *Solanum lycopersicum* (tomato)—Shivam, Arka Samrat, Arka Apeksha, and Arka Rakshak—were selected based on their agronomic performance and widespread cultivation in the local farming regions of Tamil Nadu and Karnataka. Based on prior screening for drought stress responses—including relative water content (RWC), stomatal conductance, chlorophyll fluorescence (Fv/Fm), and overall biomass retention—Shivam and Arka Samrat were identified as drought-tolerant, while Arka Rakshak and Arka Apeksha were classified as drought-susceptible genotypes (Mahapatra et al., 2025). Seeds of all genotypes were obtained from a locally situated government-certified seed vendor (Rajamanickam Seed Shop) and the Indian Institute of Horticultural Research, Bengaluru, India. The seeds were surface-sterilized with 1% sodium hypochlorite and rinsed 3–4 times with distilled water. Sterilized– seeds were sown in germination trays (protrays) filled with a 1:1:1 mixture of red soil, sand, and vermicompost. The trays were kept in a greenhouse at the School of Agriculture Innovation and Advanced Learning (VAIAL), Vellore Institute of Technology, Vellore, India, under a 12-h light/12-h dark photoperiod. The temperature was maintained at 25°C –30°C with 60%–80% relative humidity. The trays were continuously monitored, and thinning was performed as required. After 25 days of germination, at the appropriate vegetative stage, plants were grafted.

### Plant grafting and drought stress treatment

For grafting, the experiment was arranged in a completely randomized design (CRD) such that each treatment had five replicates. Six grafting combinations were established using the following combinations of rootstock and scion, respectively: G1: Shivam/Arka Samrat; G2: Shivam/Arka Rakshak; G3: Shivam/Arka Apeksha; G4: Arka Samrat/Shivam; G5: Arka Samrat/Arka Rakshak; G6: Arka Samrat/Arka Apeksha. Since rootstock plays a major role in graft compatibility and drought resistance (Razi and Muneer, 2023), the drought-tolerant genotypes Shivam and Arka Samrat were selected as rootstocks, and the drought-susceptible genotypes Arka Rakshak and Arka Apeksha were used as scions. Arka Samrat and Shivam were also homografted (G1 and G4) to assess compatibility and facilitate further observations.

The seedlings were handled for grafting at the scheduled time. Grafting was done in the early morning using the cleft-grafting method (**Supplementary Figure 1**) after reaching the vegetative stage 25 days after showing, provided the rootstock was sufficiently robust. Grafted seedlings were covered with transparent plastic to maintain 90%–95% humidity and a temperature of ~25°C–28°C, promoting graft union healing and minimizing water loss. Two weeks after grafting, when the graft junctions healed and the grafted plants survived, the uniform healthy seedlings of comparable size were planted into grow bags (two plants per bag) with the same soil combination described above. Forty days after grafting, the plants (G1-G6) were separated into two groups: control and drought stress. Drought stress was induced by gradually withholding irrigation for ten consecutive days. Soil moisture was monitored daily using a moisture meter and by weighing the grow bags. Leaf samples were collected after 10 days of treatment and stored at −80°C for further analyses.

### Morphological characteristics

All the potted crops were monitored morphologically and photographed regularly under controlled and drought-stress conditions for 10 consecutive days of treatment. The morphological parameters, including height of the plant, shoot length, and root length, were measured in centimeters by uprooting the plants carefully from the grow bags. The uprooted plants were then washed with distilled water, measured with a measuring scale, and recorded.

### Estimation of pigment contents

Total chlorophyll and carotenoid contents were determined according to Muneer et al. (2014). One gram of fresh leaf tissue was transferred to test tubes, and 5 mL of dimethyl sulfoxide (DMSO) was added to each. The tubes were then placed in a hot water bath at 65°C for 1 h to leach the pigments. After cooling, the leached pigments were measured using a UV-VIS spectrophotometer at optical densities of 480, 510, 645, and 663 nm (Sl. No. A120656, UV-1280, SHIMADZU, Japan).

Pigment contents were calculated using the formulae developed by Arnon (1949).


$$\begin{array}{c} Total\;chlorophyll\;content\;(\frac{mg}{g}freshweight)\\ =\frac{\left\{(20.2\;\text{OD}@645+8.02\;OD@663)\times V\right\}}{\left\{d\;\times 1000\times W\right\}} \end{array}$$

$$\begin{array}{c} Carotenoid\;content\;(\frac{mg}{g}freshweight)\\ =\frac{\left\{(7.6\;\text{OD}@480-1.49\;OD@510)\times V\right\}}{\left\{d\times 1000\times W\right\}} \end{array}$$

where *d* is the distance traveled by the light path in the spectrophotometer, i.e., 1 cm; *W* is the weight of the leaf sample taken in grams; and *V* is the volume of the extraction medium in milliliter.

### Lipid peroxidation level (MDA content) and proline content

For determining lipid peroxidation, also known as malondialdehyde (MDA), approximately 0.5 g of fresh samples were homogenized with 5 mL of 1% (*w*/*v*) TCA (Tricarboxylic acid) and centrifuged at 7000× *g* for 10 min (Model: ZGKU-27160, REMI NEYA 16R, REMI ELEKTROTECHNIK LTD., Vasai, India) according to Heath and Packer (1968). Following that, 1 mL of the resulting supernatant was mixed with 4 mL of a reaction solution containing 20% (*w*/*v*) TCA and 0.5% (*w*/*v*) thiobarbituric acid (TBA), then incubated in a water bath for 30 min at 95°C. The mixture was cooled instantly by placing the samples on ice. Absorbance was taken at 532 nm and 600 nm using spectrophotometer (Sl. NO-A120656, UV-1280, SHIMADZU, Japan).

For proline estimation, 0.3 g of fresh leaf tissue was homogenized in 5 mL of 3% (*w*/*v*) sulfosalicylic acid using a chilled mortar and pestle, followed by centrifugation at 3300×*g* for 20 min at 4°C. Following that, 2 mL of aliquot was mixed with 2 mL of glacial acetic acid and 2 mL of acid ninhydrin (prepared by dissolving 1.25-g acid ninhydrin in 30-mL glacial acetic acid and 20 mL of 6 N orthophosphoric acid with warming). The samples were incubated at 100°C in a water bath for 1 h. After that, the reaction was stopped by placing the tubes immediately in ice, following which 4 mL of toluene was added and vortexed. Absorbance was taken at 520 nm using a spectrophotometer (Sl. NO-A120656, UV-1280, SHIMADZU, Japan). The standard curve for proline quantification was performed according to Bates et al. (1973).

### Determination of relative water content

The relative water content (RWC) percentage was estimated according to Turner and Kramer (1978). Freshly harvested leaves were washed and weighed to obtain the fresh weight (*FW*). The leaves were then immersed in distilled water for 3 h at room temperature (25°C), and the turgid weight (*TW*) was recorded. Subsequently, the leaves were transferred to an oven (65°C) for 48 h to obtain the dry weight (*DW*). The RWC was measured using the following formula:


$$\text{RWC}\;\%=\frac{\left\{FW-DW\right\}}{\left\{TW-DW\right\}}\times 100$$

where *FW* is for fresh weight, *TW* is for turgid weight, and *DW* is for dry weight.

### Localization of stress markers H_2_O_2_ and O_2_^.-^

For H_2_O_2_ localization, fresh leaves were taken and then immersed in a 0.1% (*w/v*) solution of 3,3′-diaminobenzidine (DAB) in 5 mM of Tris-HCl buffer solution (pH 6.5) for 12 h in the dark to avoid oxidation after vacuum infiltration. Then, the leaves were submerged in 90% boiling ethanol for bleaching for 2 h in a water bath. The leaves were visualized with brown spots and documented using a digital camera (Muneer et al., 2013).

For O_2_^−^ localization, fresh leaves of equal size were taken and immersed in 0.1% solution of nitro blue tetrazolium (NBT) in 10-mM potassium phosphate buffer (pH 6.4) containing 10-mM sodium azide. The leaves were incubated in the dark for 12 h. The bleaching procedure was identical to that used for H_2_O_2_ staining. The presence of O_2_^-^ was indicated by the formation of blue formazan spots, and then, photographs were taken (Muneer et al., 2013).

### Total protein profile by SDS-PAGE

The total protein profile of all samples was determined according to Razi et al. (2021) in one dimension using sodium dodecyl sulfate-polyacrylamide gel electrophoresis (SDS-PAGE) (Razi and Muneer, 2021). The plant samples were ground in liquid nitrogen until they formed a fine powder. The extraction of protein was performed using an extraction buffer containing 40 mM (*w*/*v*) Tris-HCl, pH 7.5, 2 mM (*w*/*v*) EDTA, 2% (*w*/*v*) PVP (Polyvinylpyrrolidone), 0.07% (*v*/*v*) β-mercaptoethanol, and 1% (*v*/*v*) Triton-X-100. Then, the mixture was centrifuged at 13,000× *g* for 10 min at 4°C (Model: ZGKU-27160, REMI NEYA 16R, REMI ELEKTROTECHNIK LTD., Vasai, India). Following this, the supernatant was mixed with a protein dye containing 240 mM (*w*/*v*) Tris-HCl, pH 6.8, 8% (*w*/*v*) SDS, 40% (*v*/*v*) glycerol, 0.04% (*w*/*v*) bromophenol blue, and 5% (*v*/*v*) β-mercaptoethanol. Then, the protein samples were loaded in 12.5% polyacrylamide gel and were run in an electrode buffer using Mini PROTEAN-II (BIORAD, Hercules, CA, USA) and stained using Coomassie brilliant blue (CBB stain).

### In-gel digestion and mass spectrometer analysis

Protein bands were manually excised from the SDS-PAGE gel using a clean razor blade and rinsed three times with distilled water. The bands were further incubated for 30 min at room temperature in a de-staining solution made of 30-mM potassium ferricyanide and 100-mM sodium thiosulphate pentahydrate (1:1). After draining the de-staining solution, the gel pieces were treated with 100 mL of 50 mM NH_4_CO_3_ and then dehydrated for 5 min in 30 mL of acetonitrile.

The gel fragments were dehydrated before being subjected to 100 mL of a reduction solution (10-mM dithiothreitol in 50-mM NH_4_CO_3_) and being incubated at 56°C for 45 min. After draining the reduction solution, 100 mL of the alkylation solution was incubated at 25°C for 30 min (100-mM iodoacetamide in 50-mM NH_4_CO_3_). The gel fragments were then dehydrated for 10 min in 30 mL of acetonitrile after being rinsed in 30 mL of 50-mM NH_4_CO_3_. The gel fragments were vacuum-centrifuged, dried, and then rehydrated for 30 min at 37°C in 5–10 mL of 25-mM NH_4_CO_3_ containing 5 ng/L trypsin (Promega, Madison, WI, USA).

After 16 h of digestion at 37°C, the excess trypsin solution was replaced with 5 to 10 mL of 25-mM NH_4_CO_3_, and the digested peptides were collected, dried under vacuum, and combined with 3 L of 50% acetonitrile and 0.1% trifluoroacetic acid.

Using a mass spectrometer (Bruker Ultra flex III MALDI-TOF/TOF Mass Spectrometer, Bruker, MA, USA), materials that had been trypsin digested were examined. Subsequently, 2 mL of a 1:1 (v/v) mixture of tryptic digest and matrix solution (10 mg/ml R-cyano-4-hydroxycinnamic acid (CHCA) in 50% ACN/0.1% TFA) were spotted onto the designated plate. In total, 1,000 laser shots (summed/averaged) were used to create each mass spectrum, which was then used to gather mass spectra in reflector positive ion mode with an accelerating voltage of 21 kV spanning the mass range of 700–3,000 Da.

For external calibration, a mixture of ACTH and angiotensin standards (Sigma-Aldrich in St. Louis, MO, USA) was used. Each sample's peptide mass fingerprinting (PMFs) were chosen from monoisotopic peaks with a signal-to-noise ratio (S/N) greater than 5. For TOF/TOF fragmentation when appropriate, 2,000 laser pulses were averaged to yield parent ion spectra across a range of 40-3,000 Da.

### Functional classifications and protein–protein interaction

After the identification of proteins, their sequences were analyzed for functional classification using the online bioinformatic tool Gene Ontology (https://geneontology.org/).

Protein–protein interactions of the identified proteins were analyzed using an online bioinformatic tool -string analysis (https://string-db.org/).

### RT-PCR analysis

Total RNA isolation was performed using an RNA isolation Kit from the leaves of grafted tomato genotypes as per the manufacturer’s instructions (Hi-Media).Real-time PCR was performed in a C1000 Touch thermal cycler using SsoAdvanced Universal SYBR Green Supermix (Bio-Rad Laboratories, Inc., USA) for 3 min at 95°C, followed by 40 cycles of 10 s at 95°C, 30 s at 52°C–57°C, and following a final extension of 5 min at 72°C. Three different RNA preparations from independently grown plants were utilized for the RT-PCR reactions, along with three replicates for the qRT-PCR. The results were analyzed using qBase plus 28 software 13. The gene-specific primers used in this study are listed in **Supplementary Table S1**. These primers were designed based on gene sequences retrieved from the NCBI database and verified for specificity and purity using a genomic GC-content calculator. All primers were synthesized by Eurofins Genomics.

### Statistical analysis

Statistical analyses were performed using (SAS)-JMP PRO-17 tools (Cary, NC, USA). A completely randomized design was used with three biological replicates, and significance was set at *p* < 0.05. All results are expressed as the mean ± SE.

## Results

### Plant growth and graft junction morphology

**Figure 1:**
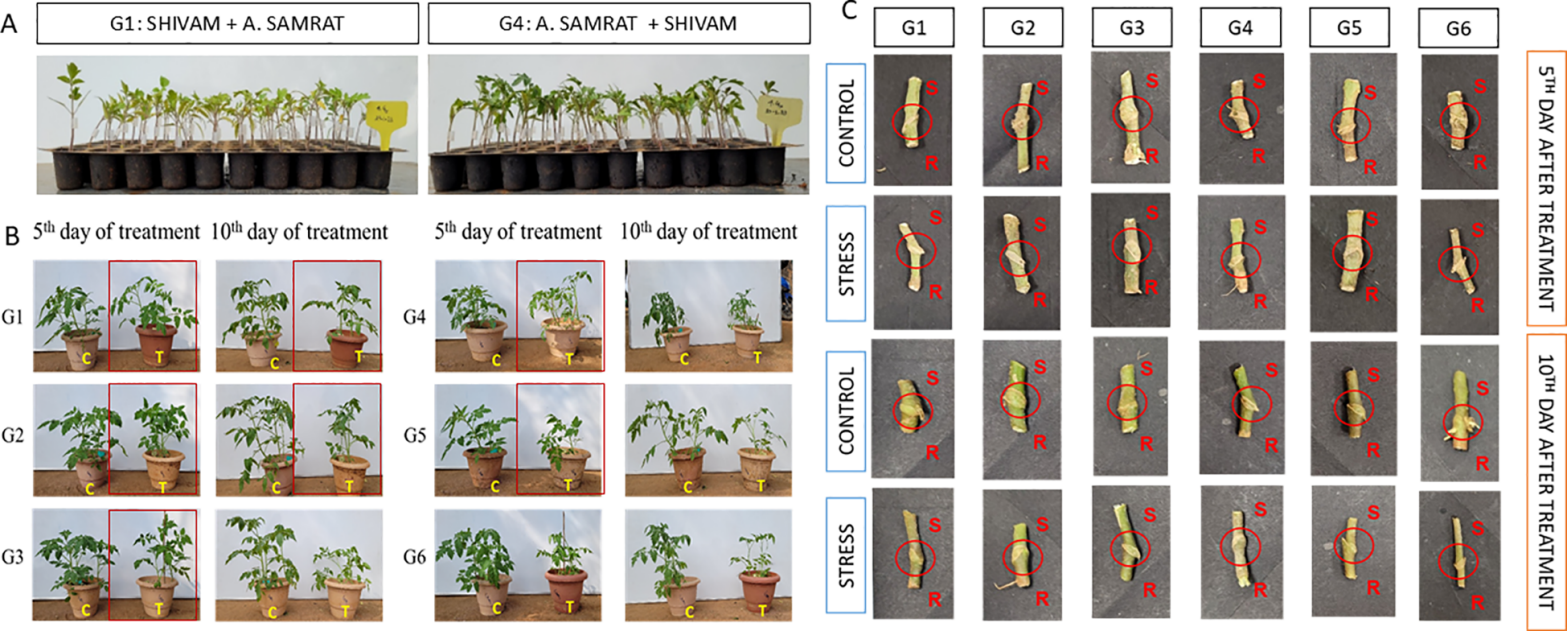
**(A)** Morphological representation of grafted tomato (*Solanum lycopersicum*) genotypes under control and drought-stressed conditions. **(B)** Graft union formation in six genotype combinations: G1: Shivam (R)/Arka Samrat (S), G2: Shivam (R)/Arka Rakshak (S), G3: Shivam (R)/Arka Apeksha (S), G4: Arka Samrat (R)/Shivam (S), G5: Arka Samrat (R)/Arka Rakshak (S), and G6: Arka Samrat (R)/Arka Apeksha (S). Photographs were taken at the vegetative stage following drought stress induction. Samples were observed after 5 days (early stage) and 10 days (late stage) of drought exposure. **(C)** indicates control and T indicates drought-treated plants. R, rootstock; S, scion.

To investigate whether the tomato genotypes Shivam and Arka Samrat, when used as rootstocks, can confer drought tolerance to drought-susceptible scion genotypes Arka Rakshak and Arka Apeksha through grafting. We made six combinations: G1: Shivam/Arka Samrat; G2: Shivam/Arka Rakshak; G3: Shivam/Arka Apeksha; G4: Arka Samrat/Shivam; G5: Arka Samrat/Arka Rakshak; and G6: Arka Samrat/Arka Apeksha. A progressive drought stress was applied to the homograft combinations for 10 consecutive days. Comparatively, all homografts remained hydrated and retained sufficient leaf foliage (**Figure 1B**). **Figure 1C** represents the morphological appearance of successfully formed grafts. The highest percentage (98%) of graft formation was observed in G1 (Shivam/Arka Samrat) and G4 (Arka Samrat/Shivam) followed by G3 (Shivam/Arka Apeksha) 85%, G2 (Shivam/Arka Rakshak) 80%, G5 (Arka Samrat/Arka Rakshak) 75%, and G6 (Arka Samrat/Arka Apeksha) 90%.

### Grafting increased photosynthetic pigments

**Figure 2:**
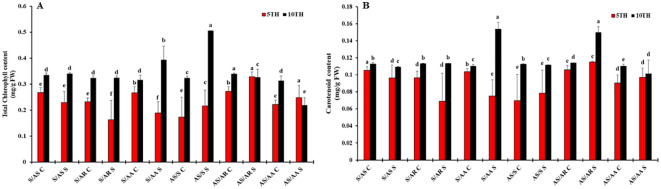
Photosynthetic pigment content in grafted tomato (Solanum lycopersicum) genotypes at the vegetative stage under drought stress. **(A)** Total chlorophyll content and **(B)** carotenoid content in six graft combinations: S/AS – Shivam (R)/Arka Samrat (S), S/AR – Shivam (R)/Arka Rakshak (S), S/AA – Shivam (R)/Arka Apeksha (S), AS/S – Arka Samrat (R)/Shivam (S), AS/AR – Arka Samrat (R)/Arka Rakshak (S), and AS/AA – Arka Samrat (R)/Arka Apeksha (S). C indicates control plants, and S indicates drought-stressed plants. Data are presented as mean ± standard error (SE), *n* = 3. Statistical significance between treatments was determined using two-way ANOVA followed by Tukey’s HSD using SAS JMP PRO 17 tools (Cary, NC, USA), with different letters indicating significant differences at *p* < 0.05.

Photosynthetic pigments play a major role in photosynthesis. We analyzed two major pigments: total chlorophyll and carotenoid content (**Figure 2**). At the early stage of drought (5 days), no significant differences were observed. However, by day 10 of drought stress, total chlorophyll content was equal to or significantly higher than that in the control homografts. This was observed in homografts of G4 (Arka Samrat/Shivam), followed by G3 (Shivam/Arka Apeksha) and G1 (Shivam/Arka Samrat) (**Figure 2A**).

Notably, carotenoid concentration in G1 and G4 was similar to the control, but G3 and G5 exhibited marginally elevated levels (**Figure 2B**). The higher carotenoid concentrations in these combinations might indicate a stress response involving main antioxidants. The findings suggest that photosynthetic activity in G4 was enhanced under drought stress, with total chlorophyll and carotenoid content aiding in the preservation of green foliage (**Figure 1**).

### Grafting maintained and enhanced proline, RWC, and MDA content

**Figure 3:**
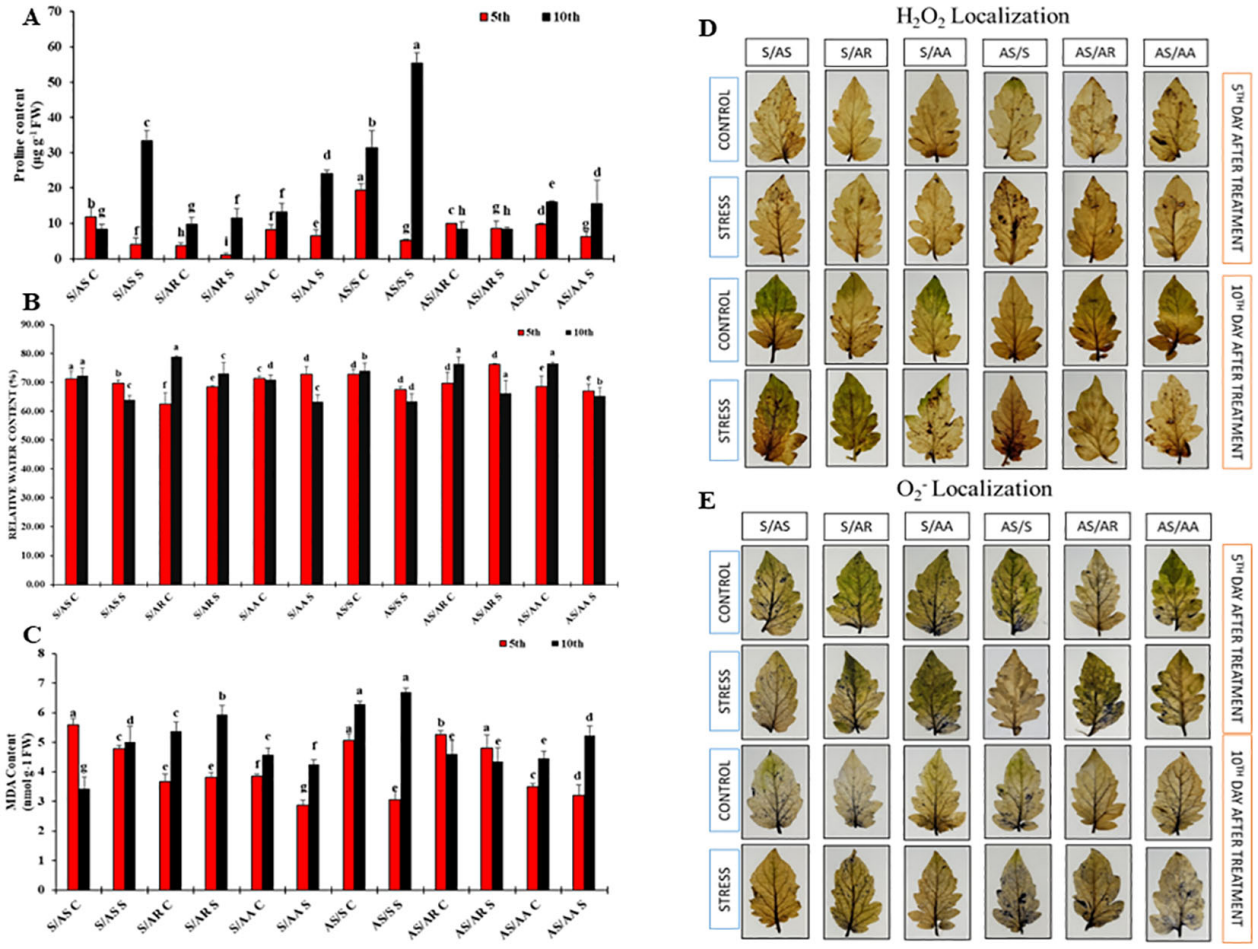
**(A)** Malondialdehyde (MDA) content, **(B)** proline content, and **(C)** relative water content (RWC) were measured at the vegetative stage in six graft combinations: S/AS – Shivam (R)/Arka Samrat (S), S/AR – Shivam (R)/Arka Rakshak (S), S/AA – Shivam (R)/Arka Apeksha (S), AS/S – Arka Samrat (R)/Shivam (S), AS/AR – Arka Samrat (R)/Arka Rakshak (S), and AS/AA – Arka Samrat (R)/Arka Apeksha (S). **(D)** Histochemical localization of hydrogen peroxide (H₂O₂) and **(E)** superoxide radicals (O₂⁻). Data for panels **(A–C)** are expressed as mean ± standard error (SE), *n* = 3. Statistical significance was assessed by one-way ANOVA followed by Tukey’s HSD test (*p* < differences among treatments. Staining intensity in panels (D, E)reflects relative levels of ROS accumulation in response to drought.

During drought stress conditions, plants often lose the ability to maintain osmotic and water potential, reducing water and nutrient absorption. To assess whether the graft combinations maintained osmoregulation, relative water content (RWC) and proline levels were measured (**Figures 3A, B**). We observed that proline content was significantly increased in drought conditions in all homograft combinations, except in G2 (Shivam/Arka Rakshak) at the early stage. Notably, G1 (Shivam/Arka Samrat combination), G4 combination (Arka Samrat/Shivam), and G3 demonstrated higher proline accumulation (Shivam/Arka Apeksha), indicating better drought resistance. Relative leaf water content was maintained at approximately 80% across all homograft combinations, even under drought conditions (**Figure 3B**).

During drought stress, reactive oxygen species (ROS) are formed, resulting in cellular damage and oxidative stress. To assess oxidative stress in drought-stressed homografts of tomato genotypes, a by-product of lipid peroxidation—malondialdehyde (MDA) content—was measured (**Figure 3C**). In all homografts, MDA content was significantly reduced by 50% at the initial stage (5 days) and increased by 20% at the later stage of drought (10 days) compared to the control. Homograft combinations such as G1 (Shivam/Arka Samrat) and G4 (Arka Samrat/Shivam) showed the highest increase in MDA content at 10 days of drought stress compared to other homografts, viz. G2 (Shivam/Arka Rakshak), G3 (Shivam/Arka Apeksha), G5 (Arka Samrat/Arka Rakshak), and G6 (Arka Samrat/Arka Apeksha).

### Grafting improved the H_2_O_2_ and O_2_ localization

Since drought stress has a greater impact on the surface of leaves in generating oxidative stress, we analyzed all tomato homografts for *in situ* localization of H_2_O_2_ and O_2_ (stress markers) (**Figures 3D, E**). Our results clearly showed negligible formation of brown and blue coloring in all homografts. In G1 (Shivam/Arka Samrat) and G4 (Arka Samrat/Shivam), H_2_O_2_ and O_2_ accumulation was limited in drought-stressed homografts compared to the control. This was followed by other homograft combinations, viz., G2 (Shivam/Arka Rakshak), G3 (Shivam/Arka Apeksha), G5 (Arka Samrat/Arka Rakshak), and G6 (Arka Samrat/Arka Apeksha). The results indicated that fewer brown and blue spots reflected minimal formation of H_2_O_2_ and O_2_ in the tissue system, providing a qualitative indication of reduced drought stress due to grafting.

### Identification of differentially expressed proteins across multiple combinations and functional annotation using gene ontology

**Figure 4:**
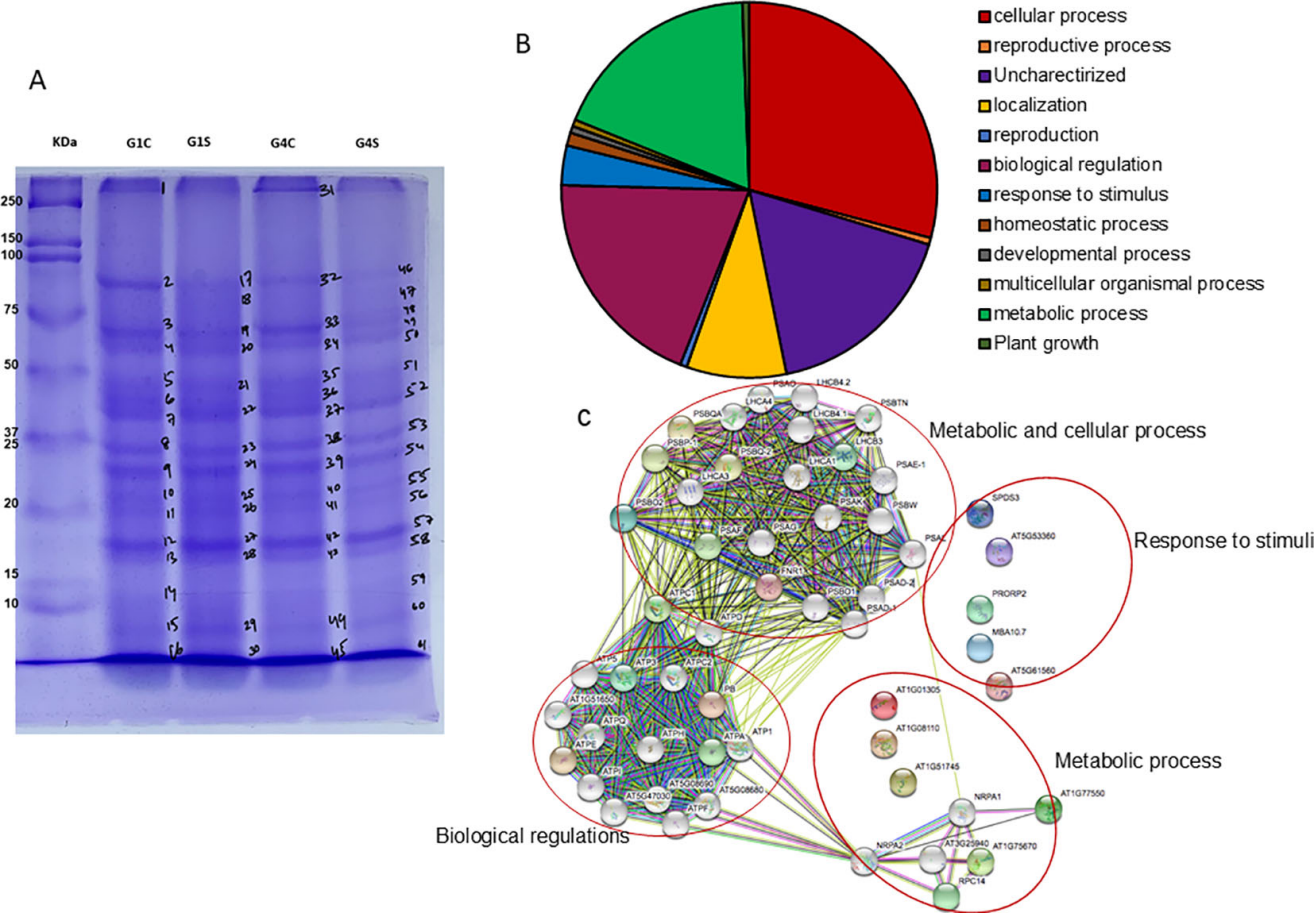
Protein profiling and interaction analysis in grafted tomato (*Solanum lycopersicum*) genotypes under drought stress. **(A)** SDS-PAGE-based protein profile of genotypes G1: Shivam (R)/Arka Samrat (S) and G4: Arka Samrat (R)/Shivam (S) at 10 days after drought stress treatment. **(B)** Pie chart representing the biological functions of differentially expressed proteins identified from drought-treated samples, categorized based on functional annotations. **(C)** Protein–protein interaction (PPI) network constructed using STRING analysis, highlighting major interaction clusters among proteins expressed in G1 and G4 under control **(C)** and drought-stressed (T) conditions. C, control; T, drought-stressed plants.

First-dimensional gel electrophoresis (SDS-PAGE) was used to analyze protein profiles and detect upregulation and downregulation of proteins (**Figure 4A**; unedited and replicate images in **Supplementary Figure 2**). These upregulated or downregulated proteins were subsequently identified using mass spectrometry (LC-MS/MS) (**Table 1**). The identified proteins were further functionally classified using gene ontology (**Figure 4B**), which highlighted a range of biological functions, reflecting the complexity of the proteomic samples. The differentially expressed proteins were associated with cellular processes (29.1%), reproductive processes (1%), uncharacterized proteins (17%), localization (8%), reproduction (1%), biological processes (19.4%), response to stimuli (1%), homeostatic processes (1%), developmental processes (1%), multicellular organismal processes (1%), metabolic processes (18%), and plant growth (1%) (**Figure 4B**). In addition, the identified proteins were evaluated for potential protein–protein interactions using the STRING database (**Figure 4C**). These included protein fragments that may act as evolutionary “bridging themes” between unrelated domains, and the wide range of isoelectric points (pI values) reflected the chemical diversity and adaptability of the proteins to various cellular environments.

**Table 1:** Identification of proteins analyzed using LC-MS/MS.

Band no.	Protein name	Origin species	Accession number	Score	Biological function	*PI* value calculated
2	Chitooligosaccharide deacetylase	*Boseongicola* sp.	A0A6L8G926	63	Catalytic activity	4
3	Uncharacterized protein	*Kalanchoe fedtschenkoi*	A0A7N0ZXR4	70	Unknown	0.79
4	ATP synthase subunit beta (Fragment)	*Didierea madagascariensis*	H6T8A3	84	Catalytic activity	0.031
5	Uncharacterized protein	*Sclerotinia borealis*	W9CKD5	60	Unknown	7.6
6	DNA-directed RNA polymerase	*Morella rubra*	A0A6A1ULK9	72	Catalytic activity	0.56
7	Tight adherence protein C	*Rhizobium* sp.	A0A4R1YKH8	64	RNA polymerase activity	3.2
8	Ferredoxin–NADP reductase, leaf-type isozyme, chloroplastic	*Nicotiana tabacum*	FENR1_TOBAC	77	Catalytic activity	0.011
9	Oxygen-evolving enhancer protein 1, chloroplastic	*Solanum lycopersicum*	P23322	69	Photosynthesis	1
10	Uncharacterized protein At1g51745	*Arabidopsis thaliana*	Y1745_ARATH	60	Unknown	0.57
11	DnaJ family molecular chaperone	*Rhizobium leguminosarum*	A0A7G6RKC7	71	Unknown	0.65
12	Type VI secretion system membrane subunit TssM	*Stenotrophomonas* sp	A0A2T3WLZ0	60	transmembrane transport	7.6
13	Orotate phosphoribosyl transferase OS	*Rhizobium leguminosarum*	A0A7W9ZRH6	70	Catalytic activity	0.72
14	U-box domain-containing protein 51	*Arabidopsis thaliana*	PUB51_ARATH	58	Catalytic activity	0.97
15	Uncharacterized protein	*Acer yangbiense*	A0A5C7ISH6	67	Unknown	1.7
16	Uncharacterized protein	*Eutrema salsugineum*	V4KDM7	73	Unknown	0.43
49	E3 ubiquitin-protein	*Fusarium longipes*	A0A395SD28	61	Uncharacterized	6.3
50	ATP synthase subunit beta, chloroplastic	*Calystegia sepium*	ATPB_CALSE	66	Catalytic activity	0.15
51	Uncharacterized protein	*Pseudomonas brassicacearum*	A0A423GJS7	62	Unknown	4.8
53	Ferredoxin–NADP (+) reductase (Fragment)	*Solanum pimpinellifolium*	Q30GR3	86	Catalytic activity	0.022
54	DDE family transposase	*Paraburkholderia sediminicola*	A0A371DTU3	66	Transposon/transposable element	2.2
56	Proteinaceous RNase P 2	*Arabidopsis thaliana*	PRRP2_ARATH	64	Catalytic activity	0.24
57	Uncharacterized protein	*Setaria italica*	A0A368RM25	67	Unknown	1.7
58	Ectoine/hydroxyectoine ABC transporter ATP-binding protein EhuA	*Rhizobium meliloti*	A0A222JJ69	65	Transmembrane transport	2.5
59	Spermine/spermidine synthase	*Bradyrhizobium daqingense*	A0A562LUJ0	69	Hydrolase activity	1.1
60	Tubulin-tyrosine ligase	*Polyporus brumalis*	A0A371DTU3	62	Ligase activity	5.4
61	Lactoylglutathione lyase	*Noccaea caerulescens*	A0A1J3I8A8	75	Catalytic activity	0.28

### Protein–protein interactions

The protein–protein interaction (PPI) network generated using STRING 11.5 revealed functional relationships among the proteins identified by LC-MS/MS in grafted tomato genotypes under drought stress (**Figure 4C**). Key clusters of interactions are highlighted with red-colored circles. The PPI network demonstrated major groups of proteins associated with metabolic and cellular processes, as well as biological regulation. STRING analysis of the grafted genotypes showed a particularly high level of interaction among plant photosynthesis proteins, compared to other functional categories. This finding reveals that grafting enhances protein interactions, forming a “buffer system” whereby rootstock-derived signals strengthen scion protein networks involved in stress perception (e.g., ABA receptors), damage prevention (e.g., HSPs), and recovery (e.g., photosynthesis proteins). The integration of mobile mRNAs with RNA-binding proteins (RBPs) and epigenetic modifiers represents a novel layer of post-transcriptional regulation in grafted responses to drought. The PPI results also depict a greater percentage of proteins related to stress responses and metabolic processes, which could help cell division and growth even under drought stress.

### Relative gene expression

To confirm our findings, we performed quantitative real-time PCR (qRT-PCR) for G1 (Shivam/Arka Samrat) and G4 (Arka Samrat/Shivam). Genes were selected based on their roles in responding to environmental stress, such as drought and heat, including hormonal changes, regulation of transcription factors for abiotic stress, and enzymatic–antioxidant functions in grafted tomato plants.

**Figure 5:**
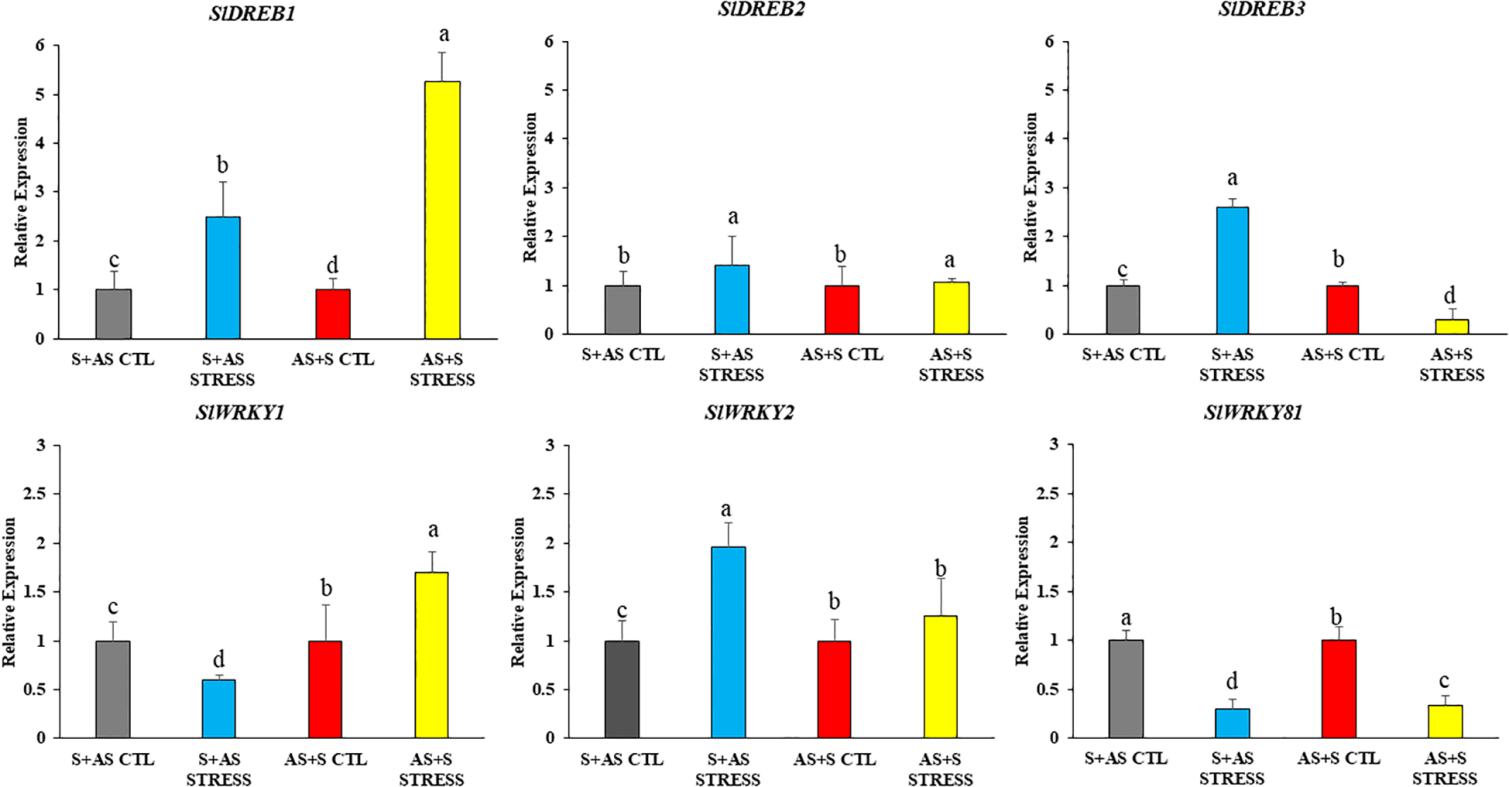
Relative expression of drought-responsive genes (*SlDREB1*, *SlDREB2, SlDREB3, SlWRKY1, SlWRKY2, SlWRKY81*) in grafted tomato (*Solanum lycopersicum*) genotypes at 10 days after drought treatment. G1 (S+AS): Shivam/Arka Samrat; G4 (AS+S): Arka Samrat/Shivam under control (CTL) and drought stress (STRESS). Data are expressed as fold change relative to control and presented as mean ± SE (*n* = 3). Statistical significance was determined by two-way ANOVA followed by Tukey’s HSD (*p* < 0.05), using (SAS)-JMP PRO-17 tools (Cary, NC, USA); different letters indicate significant differences.

We first examined the expression levels of drought-responsive genes (**Figure 5**). Genes including *SlDREB1*, *SlDREB2*, *SlDREB3*, *SlWRKY1*, *SlWRKY2*, and *SlWRKY81* showed a significant increase of up to threefold at the early stage of drought stress, except *SlWRKY1* and *SlWRKY2*. Expression levels further increased to 1–5-fold at later stages of drought stress.

**Figure 6:**
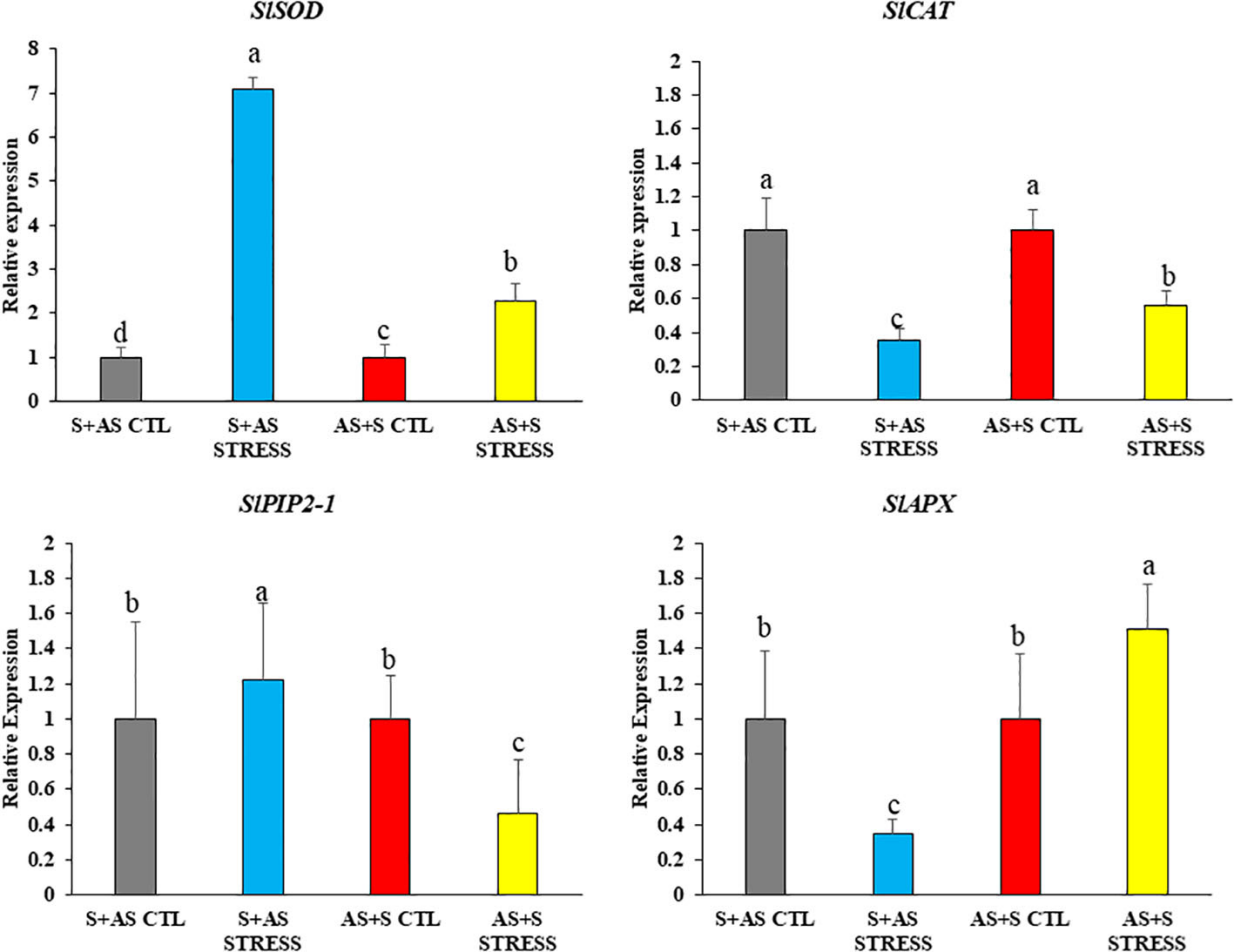
Relative gene expression of antioxidant response genes (*SlSOD*, *SlCAT*, *SlAPX*) and aquaporin genes; *SlPIP2-1*) in grafted tomato (*Solanum lycopersicum*) genotypes at 10 days after drought treatment. G1 (S+AS): Shivam/Arka Samrat; G4 (AS+S): Arka Samrat/Shivam under control (CTL) and drought stress (STRESS). Data are expressed as fold change relative to control and presented as mean ± SE (*n* = 3). Statistical significance was determined by two-way ANOVA followed by Tukey’s HSD (*p* < 0.05), using (SAS)-JMP PRO-17 tools (Cary, NC, USA); different letters indicate significant differences.

Genes related to oxidative stress and aquaporin activity, such as *SlSOD*, *SlCAT*, *SlPIP2–1*, and *SlAPX*, were analyzed to assess how grafting influenced their involvement in mitigating oxidative stress (**Figure 6**). *SlSOD* and *SlPIP2–1* were significantly upregulated by 6-fold and 1.2-fold, respectively, in G1 (Shivam/Arka Samrat) and G4 (Arka Samrat/Shivam) compared to their respective controls under drought stress, although *SlPIP2–1* showed variation *–* in G4 (Arka Samrat/Shivam) plants (**Figure 6**). The relative expression of *SlCAT* and *SlAPX* decreased significantly (to 0.5-fold) in G1, while their expression (Shivam/Arka Samrat) increased up to fivefold in G4 (Arka Samrat/Shivam).

**Figure 7:**
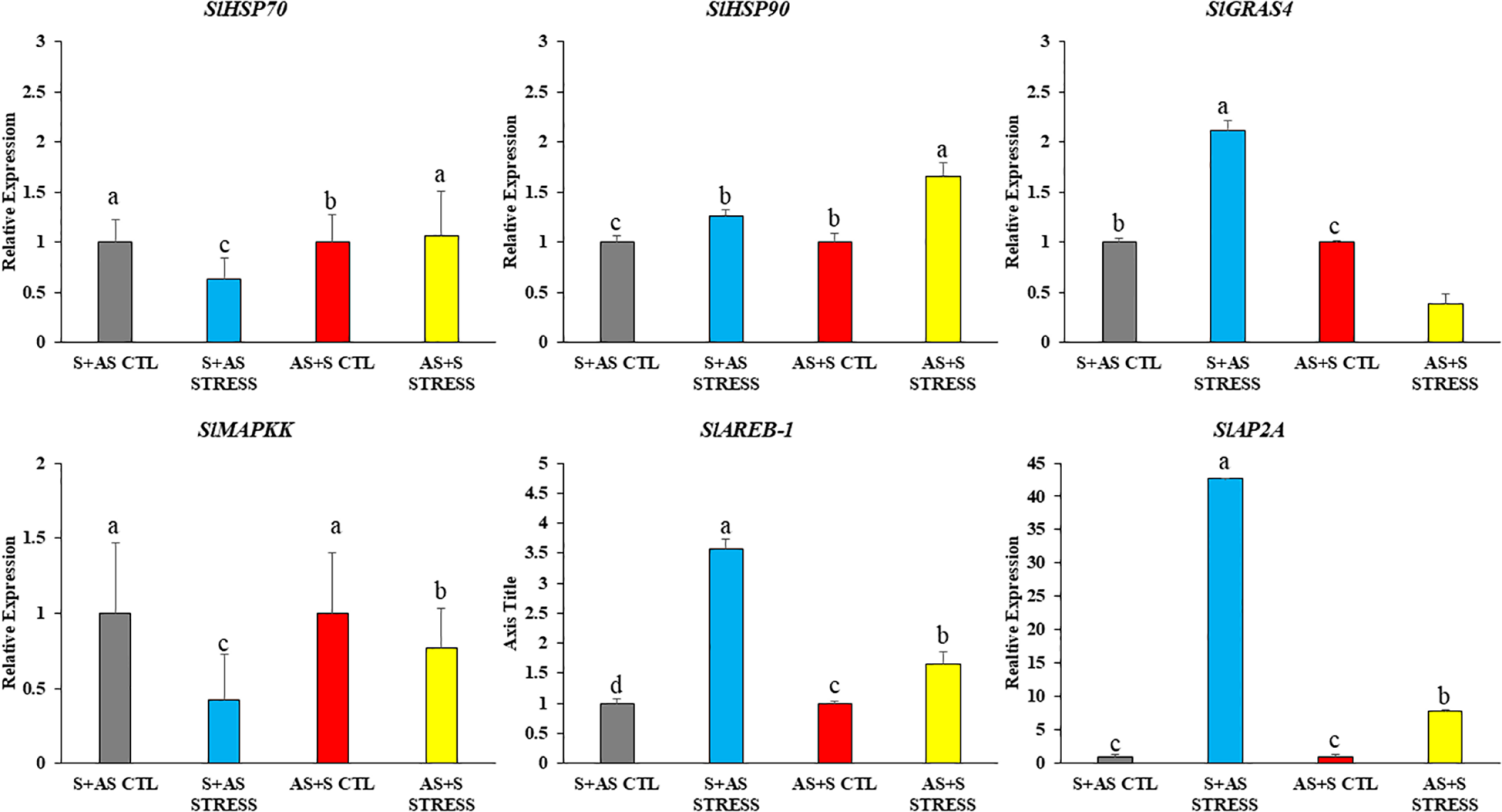
Relative gene expression of heat shock and other stress-responsive genes (*SlHSP70*, *SlHSP90*, *SlGRAS4, SlMAPKK, SlAREB-1, SlAP2a*) in grafted tomato (*Solanum lycopersicum*) genotypes at 10 days after drought treatment. G1 (S+AS): Shivam/Arka Samrat; G4 (AS+S): Arka Samrat/Shivam under control (CTL) and drought stress (STRESS). Data are expressed as fold change relative to control and presented as mean ± SE (*n* = 3). Statistical significance was determined by two-way ANOVA followed by Tukey’s HSD test (*p*< 0.05), using (SAS)-JMP PRO-17 tools (Cary, NC, USA); different letters indicate significant differences.

We also analyzed the expression levels of genes related to heat shock and MAP kinase pathways due to their direct involvement in drought stress (**Figure 7**). For *SlHSP70* and *SlHSP90*, there was a significant difference between the control and drought-stressed genotypes of grafted tomatoes. In G1 (Shivam/Arka Samrat), *SlHSP70* showed significantly lower expression, while *SlHSP90* exhibited higher expression levels compared to G4 (Arka Samrat/Shivam), which was concomitantly expressed in higher levels. *SlGRAS4* in G1 (Shivam/Arka Samrat) showed a one-fold increase compared to the control whereas in G4 (Arka Samrat/Shivam). it decreased to 0.5-fold. *SlMAPKK* showed a significantly lower expression in stress conditions compared to control for both G1 (Shivam/Arka Samrat) and G4 (Arka Samrat/Shivam). Genes such as *SlAREB-1* and *SlAP2a* showed a significantly higher expression in drought-stressed plant for G1 (Shivam/Arka Samrat) compared to G4 (Arka Samrat/Shivam).

**Figure 8:**
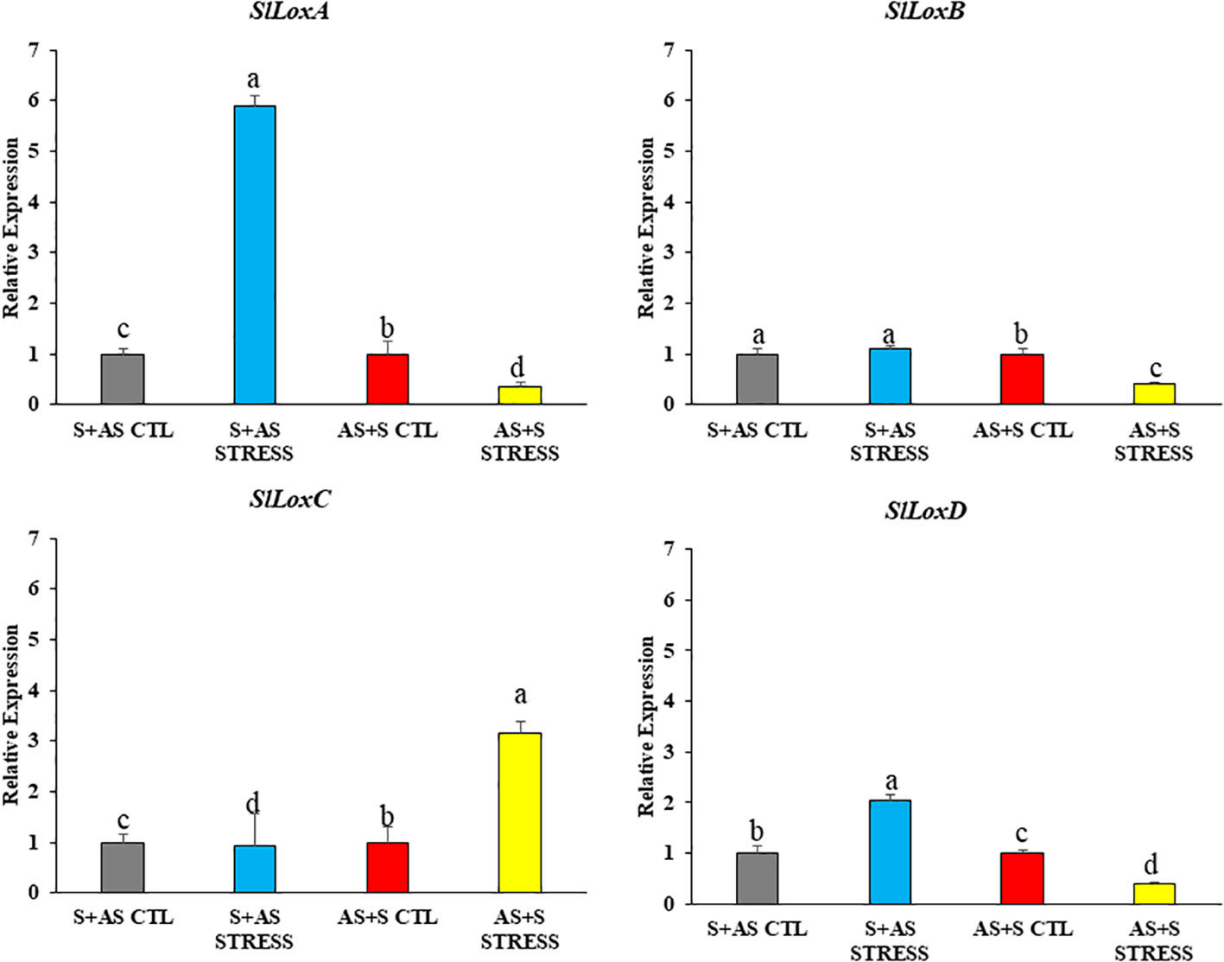
Relative expression of LOX genes (*SlLoxA*, *SlLoxB*, *SlLoxC*, *SlLoxD*) in grafted tomato (*Solanum lycopersicum*) genotypes 10 days after drought treatment. G1 (S+AS): Shivam/Arka Samrat; G4 (AS+S): Arka Samrat/Shivam under control (CTL) and drought stress (STRESS). Data are expressed as fold change relative to control and presented as mean ± SE (*n* = 3). Statistical significance was determined by two-way ANOVA followed by Tukey’s HSD (*p* < 0.05), using (SAS)-JMP PRO-17 tools (Cary, NC, USA); different letters indicate significant differences.

Lipoxygenase (LOX) genes, which are involved in drought defense, were also analyzed (**Figure 8**). In G1 (Shivam/Arka Samrat) drought-stressed plants showed significantly higher expression of *SlLoxA*, whereas in G4,(Arka Samrat/Shivam) the expression was reduced. Genes like *SlLoxB* and *LoxD* showed similar expression levels in G4 (Arka Samrat/Shivam), while G1 (Shivam/Arka Samrat) displayed lower fold change under drought stress compared to control. For *SlLoxC*, there was no difference between stress and control in G1 (Shivam/Arka Samrat); however, in G4 (Arka Samrat/Shivam), expression was twofold higher under stress (**Figure 8**).

## Discussion

Drought greatly influences plant growth and development, and it is essential to know how to mitigate it through grafting (Zia et al., 2021). Grafting improves plant drought resistance (Chen et al., 2020; Razi and Muneer, 2023) and is primarily determined by the rootstock (Han et al., 2020; Chen et al., 2020; López-Hinojosa et al., 2021). Therefore, the current study has shown how selected rootstocks and grafting can mitigate drought stress effects on tomato by linking physiology and molecular mechanisms, including proteome and transcriptome. The root is an important organ for water and nutrient uptake to support growth and development, and graft compatibility is one of the crucial factors. Drought usually reduces root biomass, damages root systems, and decreases root hydraulic pressure (Yang et al., 2022; Zia et al., 2021).

In our study, we first selected several combinations of tomato genotypes for grafting to check compatibility (rootstock/scion) (**Figure 1**), viz., G1: Shivam/Arka Samrat; G2: Shivam/Arka Rakshak; G3: Shivam/Arka Apeksha; G4: Arka Samrat/Shivam; G5: Arka Samrat/Arka Rakshak; G6: Arka Samrat/Arka Apeksha. All selected graft combinations of tomato genotypes showed compatibility with each other, but greater compatibility was observed for G1: Shivam/Arka Samrat and G4: Arka Samrat/Shivam.

Scions maintain their own growth and development through photosynthetic products such as pigments present in the leaf. Drought stress induced adverse effects on scions like wilting, leaf area reduction (**Figure 1B**), reduced leaf and stem biomass, and weakened photosynthetic pigments (**Figure 2**). Grafting onto drought-resistant rootstocks G1: Shivam/Arka Samrat and G4: Arka Samrat/Shivam showed greater photosynthetic pigment content compared to drought-sensitive rootstocks G2: Shivam/Arka Rakshak, G3: Shivam/Arka Apeksha, G5: Arka Samrat/Arka Rakshak, and G6: Arka Samrat/Arka Apeksha. This indicates that grafting onto a drought-resistant rootstock can be a good strategy to alleviate negative effects (Shehata et al., 2022), as also observed in several fruit crops such as grapevine (Sucu et al., 2018), apple (Valverdi and Kalcsits, 2021), and watermelon (Ali et al., 2019; Bikdeloo et al., 2021; Parsafar et al., 2019). In other studies, scions grafted on drought-resistant tomato rootstocks depicted lower photosynthesis (Alves et al., 2021). The studies on photosynthetic pigments provide a basis for high CO_2_ assimilation under drought-stressed conditions in grafted tomato genotypes, particularly with G1 and G4 where resistant genotypes were used as rootstocks (**Figure 2**).

Drought stress causes osmotic stress in plant cells, leading to ion imbalance and relative loss of water content in plants (Zia et al., 2021; Sánchez-Rodríguez et al., 2012). Under low osmotic potential, osmoregulation of grafted plants depends on active compounds that can improve water retention (Ozturk et al., 2021), as also shown by our results of relative water potential (**Figure 3B**). Proline is one of the osmoprotectants that can stabilize membrane and protein formation. Proline was found to accumulate profusely in all tomato genotypes under drought stress and in higher quantities in G1: Shivam/Arka Samrat and G4: Arka Samrat/Shivam compared G2: Shivam/Arka Rakshak; G3: Shivam/Arka Apeksha; G5: Arka Samrat/Arka Rakshak; and G6: Arka Samrat/Arka Apeksha (**Figure 3A**). This result indicates that drought-resistant genotypes are better suited as rootstocks and susceptible ones as scions. Proline accumulation in plant tissues in higher quantities can be achieved due to activation of proline biosynthesis or oxidative stress inhibition (Szabados and Savouré, 2010).

Our results for oxidative stress markers such as MDA content and the relative localization of H_2_O_2_ and O_2_ (**Figures 3C, D**) depicted that grafting can reduce oxidative damage accumulation at the cellular level. Oxygen radicals abundantly accumulated in all tomato genotypes under drought stress but were present in lower quantities in G1: Shivam/Arka Samrat and G4: Arka Samrat/Shivam compared to G2: Shivam/Arka Rakshak; G3: Shivam/Arka Apeksha; G5: Arka Samrat/Arka Rakshak; and G6: Arka Samrat/Arka Apeksha. This could have been due to the activation of antioxidative enzyme defense mechanisms as shown in our transcriptional studies (**Figure 6**), to scavenge ROS, as also previously observed in our okra studies (Razi and Muneer, 2023).

In several grafted plants such as poplar, tomato, and citrus, higher soluble protein content was found under drought stress, which contributed to strong osmotic adjustment (Han et al., 2019; Zhang et al., 2020; Dong et al., 2023). For instance, late embryogenesis abundant (LEA) proteins and osmotins are common proteins that stabilize cell membranes and promote osmoregulation during drought stress (Ozturk et al., 2021). We identified several proteins (**Table 1**) involved in various molecular mechanisms (**Figure 4B**) under drought stress in different grafting combinations, -G1: Shivam/Arka Samrat and G4: Arka Samrat/Shivam compared G2: Shivam/Arka Rakshak; G3: Shivam/Arka Apeksha; G5: Arka Samrat/Arka Rakshak; and G6: Arka Samrat/Arka Apeksha. For instance, proteins identified from grafted tomato genotypes were primarily related to biological regulations such as catalytic activity (band numbers 8, 13, 14, 50, 53, 56, and 61). One identified protein related to hydrolase activity (band number 59) indicated that this protein promoted osmoregulation. Proteins such as Type VI secretion system membrane subunit TssM (band 12) and ectoine/hydroxyectoine ABC transporter ATP-binding protein EhuA (band 58) indicated that grafting helped drought-stressed tomatoes maintain water content. Similarly, the protein identified as tubulin-tyrosine ligase (band 60) suggested lignification under drought stress in grafted plants.

The overall proteins identified indicate that grafting helped all tomato genotypes in this experimental study to overcome many negative cellular modifications by improving proteins involved in biological processes such as catalytic activity, photosynthesis, homeostatic maintenance, lignification, and response to stimuli, as also confirmed by protein–protein interactions (**Figure 4C**).

DREB genes play an important role in ABA-independent synthesis pathways. Several studies have focused on the regulation of DREB transcription factor expression under various abiotic stresses (Zhang and Xia, 2023). The expression and regulation network of DREB transcription factors under abiotic stress is complex, but it is crucial for improving plant stress resistance. In combination with relevant reports on DREB genes in the past decade, this study analyzed the participation of DREB genes such as *SlDREB1*, *SlDREB2*, and *SlDREB3*.

Since then, DREBs have been reported to be involved in various abiotic stress responses through both ABA-dependent and ABA-independent pathways (Yamaguchi-Shinozaki and Shinozaki, 1994; Busk et al., 1997; Liu et al., 1998; Kizis and Pagès, 2002; Dubouzet et al., 2003). Our study showed that all the transcription factors of DREB genes contributed to improved drought stress tolerance in grafted tomatoes, particularly in the G1 and G4 combinations where resistant genotypes were used as rootstock and scion.

WRKY transcription factors are also involved in the drought stress response (Rushton et al., 2010; Chen et al., 2019), regulating drought tolerance via abscisic acid (ABA) signaling pathways (Rushton et al., 2012). It has been observed that AtWRKY57 improves drought tolerance in Arabidopsis by binding to W-box elements to activate the expression of RD29A and NCED3 (Jiang et al., 2012). Our studies on WRKY genes, such as *SlWRKY1*, *SlWRKY2*, and *SlWRKY81*, also indicated their involvement in regulating drought stress in grafted tomato plants.

The expression of DREB and WRKY transcription factors suggests their active participation in drought response mechanisms in tomato genotypes of the G1: Shivam/Arka Samrat and G4: Arka Samrat/Shivam graft combinations. These findings are supported by our transcriptional analysis of antioxidative genes such as *SlSOD*, *SlAPX*, and *SlCAT* (**Figure 6**).

Additionally, our transcriptional studies on *SlPIP1*, an aquaporin gene, further indicated that grafted plants were able to maintain osmotic potential under drought conditions. This is consistent with our previous findings in okra (Razi and Muneer, 2023) and in pearl millet (Iwuala et al., 2020).

Heat shock proteins (HSPs) have been identified to increase dramatically when cells are exposed to high temperatures. As molecular chaperones, HSPs play critical roles in acquired thermotolerance under heat stress and act as negative feedback regulators of heat shock transcription factor (HSF) activity (Kim and Schöffl, 2002; Yang et al., 2023). In this study, HSP70 and HSP90 were found to be upregulated under drought stress, contributing to homeostatic maintenance in the G1: Shivam/Arka Samrat and G4: Arka Samrat/Shivam combinations.

This finding was further supported by other transcription factor genes such as *SlGRAS4*, *SlMAPKK*, *SlAREB-1*, and *SlAP2a*, which are known to modulate drought stress responses in several crops including wheat (Mishra et al., 2023), rice (Ning et al., 2011), sandalwood (Meng et al., 2024), and Populus (Kong et al., 2023).

Lipoxygenase (LOX) gene families have been increasingly studied for their diverse roles in plant stress responses (Li W. et al., 2022). In Arabidopsis, *lox3 lox4* double mutants showed defective global proliferative arrest, and these genes have been linked to resistance against plant-parasitic nematodes (Ozalvo et al., 2014). *AtLOX2* and *AtLOX6* are involved in jasmonic acid (JA) biosynthesis and are induced by different stresses (Bell et al., 1995; Grebner et al., 2013).

In addition, rice OsHI-LOX, maize ZmLOX10, and tobacco NaLOX3 mediate herbivore-induced defense and are all involved in JA biosynthesis (Zhou et al., 2009; Allmann et al., 2010; Christensen et al., 2013). Furthermore, in maize, *ZmLOX10* was found to localize to organelles and modulate both direct and indirect defenses against herbivores (Christensen W. et al., 2013; Liu et al., 2021; Zhou et al., 2013).

Our findings are consistent with previous studies which proved that LOX genes play multiple roles in cotton (Shaban et al., 2018), tomato (Upadhyay et al., 2019), and peanut (Mou et al., 2022). A recent study demonstrated that cis-acting elements in LOX genes regulate responses to abiotic stresses in tomato (Halitschke and Baldwin, 2003; Upadhyay et al., 2019). In addition, two previous studies revealed that the LOX gene played an important role in resistance to root-knot nematodes (Gao et al., 2009) and phloem feeders (Zhou et al., 2009).

Altogether, this analysis further supports the conclusion that grafting enhances the activation of LOX genes involved in defense responses to drought stress in tomato genotypes, particularly when drought-resistant genotypes are used as rootstocks.

## Conclusions

Grafting is an important agricultural practice for enhancing tolerance to both biotic and abiotic stress, and the selection of suitable rootstocks and scions is crucial for conferring drought resistance in grafted plants. Moreover, grafted plants regulate morphological, physiological, and molecular responses that enable them to adapt to drought stress.

In this study, we observed a comparative increase in physiological, morphological, and molecular traits, with G1 and G4 combinations showing good performance, followed by G3, G5, G6, and G2. Graft-responsive proteins, along with drought-inducible genes and those involved in rootstock–scion communication, contributed significantly to improved drought tolerance (Schwarz et al., 2013).

**Figure 9:**
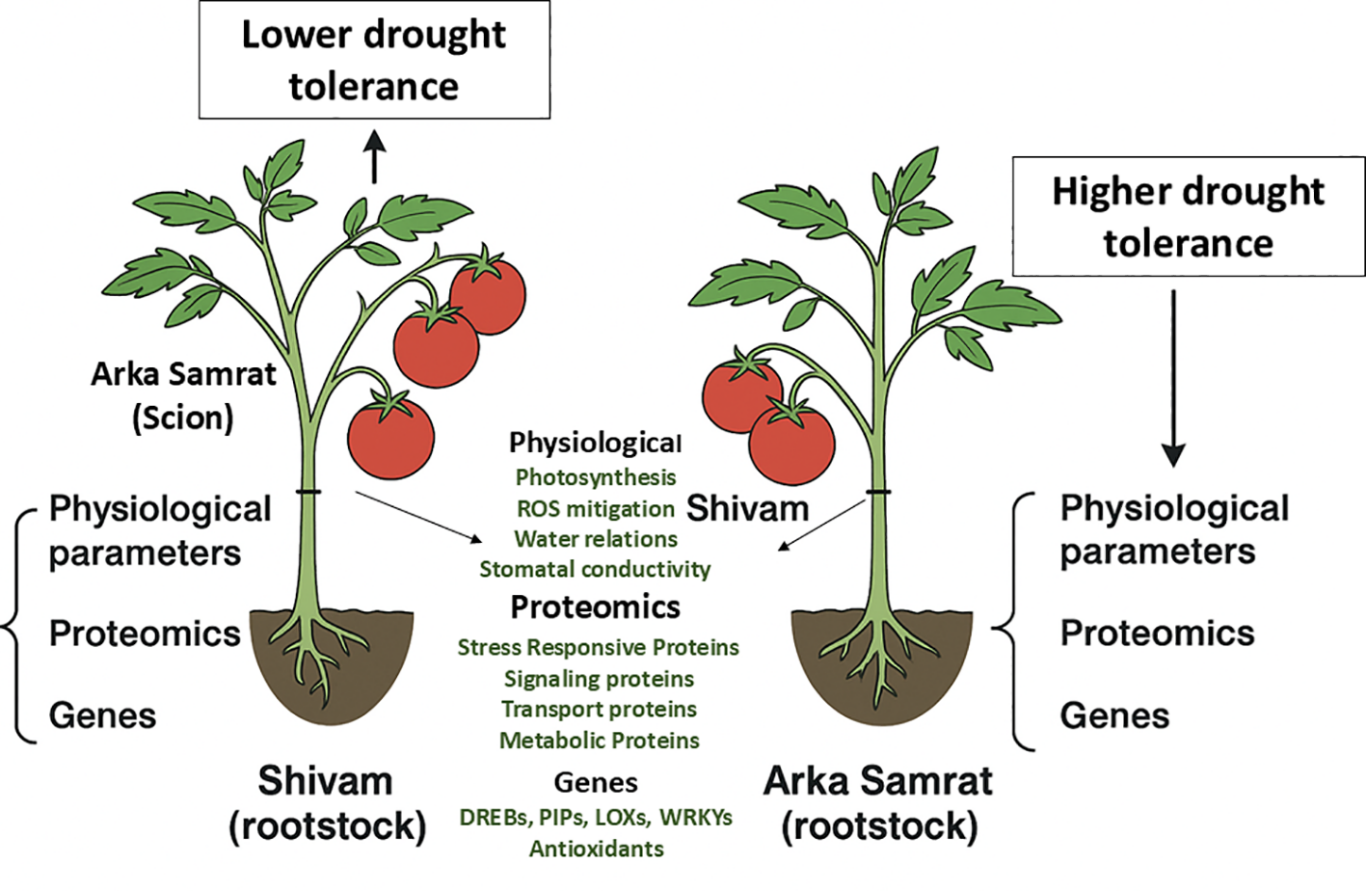
Schematic overview of the effect of rootstock on scion drought tolerance in grafted tomato plants. Tomato genotypes Shivam and Arka Samrat were reciprocally grafted to evaluate physiological, proteomic, and gene expression responses under drought. The figure summarizes key rootstock-induced improvements in the scion, including enhanced water relations, upregulation of stress-related proteins, and differential expression of drought-responsive genes.

However, key transcription factors associated with oxidative stress responses—including *DREB*, *WRKY*, *AREB1*, *GRAS4*, and *AP2a*—showed relatively lower fold increases under drought conditions. This suggests that their expression may be tightly regulated by specific graft combinations and underlying signaling mechanisms (**Figure 9**).

Furthermore, selecting drought-resistant genotypes for both rootstock and scion is essential for improving drought tolerance, especially in crops grown in arid and dry regions. As grafting remains a complex biological process, future research into graft healing and the integration of artificial intelligence holds promise for advancing our understanding and enhancing the success of grafting under drought stress.

## Data Availability

The datasets presented in this study can be found in online repositories. The names of the repository/repositories and accession number(s) can be found in the article/**Supplementary Material**.
