# Correction: Grafting enhances drought stress tolerance by regulating the proteome and targeted gene regulatory networks in tomato

**DOI:** 10.3389/fpls.2025.1692342

**Published:** 2025-10-03

**Authors:** Pritam Paramguru Mahapatra, Dong Won Bae, Michitaka Notaguchi, Sowbiya Muneer

**Affiliations:** ^1^ Horticulture and Molecular Physiology Lab, Department of Horticulture and Food Science, School of Agricultural Innovations and Advanced Learning, Vellore Institute of Technology, Vellore, Tamil Nadu, India; ^2^ School of Biosciences and Technology, Vellore Institute of Technology, Vellore, Tamil Nadu, India; ^3^ Central Instrument Facility, Gyeongsang National University, Jinju, Republic of Korea; ^4^ Department of Botany, Graduate School of Science, Kyoto University, Kyoto, Japan

**Keywords:** drought, gene-regulatory network, proteome, resistant, stress tolerance, tomato

In the published article, Equation in Section- Materials and methods, subsection- *Estimation of pigment contents* after 2^nd^ paragraph was erroneously given as


$$“\text{RWC}\;\%=\frac{\left\{FW-DW\right\}}{\left\{TW-DW\right\}}\times 100”$$

.

The correct equation is


$$\begin{array}{c} “Total\;chlorophyll\;content\;(\frac{mg}{g}freshweight)\\ =\frac{\left\{(20.2\;\text{OD}@645+8.02\;OD@663)\times V\right\}}{\left\{d\;\times 1000\times W\right\}}“ \end{array}$$

$$“Carotenoid\;content\;(\frac{mg}{g}freshweight)=\frac{\left\{(7.6\;\text{OD}@480-1.49\;OD@510)\times V\right\}}{\left\{d\times 1000\times W\right\}}”$$

Equation in Section- Materials and methods, subsection- *Determination of relative water content* after 1^st^ paragraph was erroneously given as


$$\begin{array}{c} “Total\;chlorophyll\;content\;(\frac{mg}{g}freshweight)\\ =\frac{\left\{(20.2\;\text{OD}@645+8.02\;OD@663)\times V\right\}}{\left\{d\;\times 1000\times W\right\}}” \end{array}$$

$$\begin{array}{c} “Carotenoid\;content\;(\frac{mg}{g}freshweight)\\ =\frac{\left\{(7.6\;\text{OD}@480-1.49\;OD@510)\times V\right\}}{\left\{d\times 1000\times W\right\}}” \end{array}$$

where *FW* is for fresh weight, *TW* is for turgid weight, and *DW* is for dry weight.


$$“\text{RWC}\;\%=\frac{\left\{FW-DW\right\}}{\left\{TW-DW\right\}}\times 100”$$

The correct equation is


$$“\text{RWC}\;\%=\frac{\left\{FW-DW\right\}}{\left\{TW-DW\right\}}\times 100”$$

where *FW* is for fresh weight, *TW* is for turgid weight, and *DW* is for dry weight.

The original version of this article has been updated.

