# Supplementary material for: Grafting enhances drought stress tolerance by regulating the proteome and targeted gene regulatory networks in tomato
Source: Front Plant Sci. 2025 Aug 20;16:1591437. doi: 10.3389/fpls.2025.1591437 (PMC12405251; doi:10.3389/fpls.2025.1591437)
Supplement: Supplementary file 1 [file Table1.docx]

|  | **Forward** | **Reverse** |
| --- | --- | --- |
| *DREB1* | CACCGTTGCTTCTGGTTTCG | TTCTTGCCTGCCTGGTTTCA |
| *DREB2* | GGGAGCCACATAGAGGGAGA | ATCGGCATTGTCCAACTGGT |
| *DREB3* | CTTTGGCTTGGTACATTTG | GCAATGGATTGTATTCACC |
| *WRKY1* | CAAAGAGACGGAAGAAAGA | GACTACATCCACTTCACTG |
| *WRKY2* | GAATCAGGTGATGCAGTAG | CCCTCATAACCTTGTGATAC |
| *WRKY81* | GAAGAGGATGCTACAAGAG | GTAGTGCCTTGGATAAGTAG |
| *AREB1* | GGCAGCCATCTATCTATTC | TTGATTCTCCTCAGCATTC |
| *AP2a* | ATATGATAGGGCAGCAATC | CCAGTACTTTGTCTCCTTAG |
| *GRAS4* | GTGCATGTACTAAGGAGAC | CATTCTAGCTTCCCATCTAC |
| *MAPKK* | CTCTGAAGGTTATCCAGATG | GTGATAGAAAGAGTGGTAGC |
| *SOD* | ACATACAAAAATGGTGAAGGCC | AGGATTGTAATGTGGTCCTGTT |
| *CAT1* | CGCATACGACACCCCTTTCT | TCGACCAACTGGGATCAACG |
| *APX* | GGATGGATGTGTCCGTACCTG | TAGTTCGTCGGGAGAGGCTT |
| *HSP70* | CGGAGGTCGATGAAGAGTCG | CCACCCAGAGTTTCCAGACC |
| *HSP90* | CAAGGAGGACTACAACAAG | GGTTGACTGATATCGAAGG |
| *PIP2-1* | CTGCGGGTATTTCTGGTGGA | CAATGGGTAGAGGCGCAAGT |
| *LoxA* | GCGTGGGATAGGATTTATG | TCTACCAGTTCTTCCTCTC |
| *LoxB* | GACAAGAGCTCAGAGATTG | CTTAAAGTAGGGCGATTAGG |
| *LoxC* | GAGGACTTGATGGTATTGG | CGATGTGCATATGACCTAA |
| *LoxD* | GAGGGAGCTAAGAGAATTG | CCTTCTCCTCCAAGTTTAG |
| *ACTIN* | GGCACCCCTTAATCCCAAGG | GCTGGAATGTGCTGAGGGAT |

**Table 1:** List of primer sequences utilized for the gene expression analyses through RT-PCR analysis. It consists of set of gene name and their sequences which were calculated using Actin-1 (Internal control).
