# Supplementary figures and images for: Grafting enhances drought stress tolerance by regulating the proteome and targeted gene regulatory networks in tomato
Source: Front Plant Sci. 2025 Aug 20;16:1591437. doi: 10.3389/fpls.2025.1591437 (PMC12405251; doi:10.3389/fpls.2025.1591437)

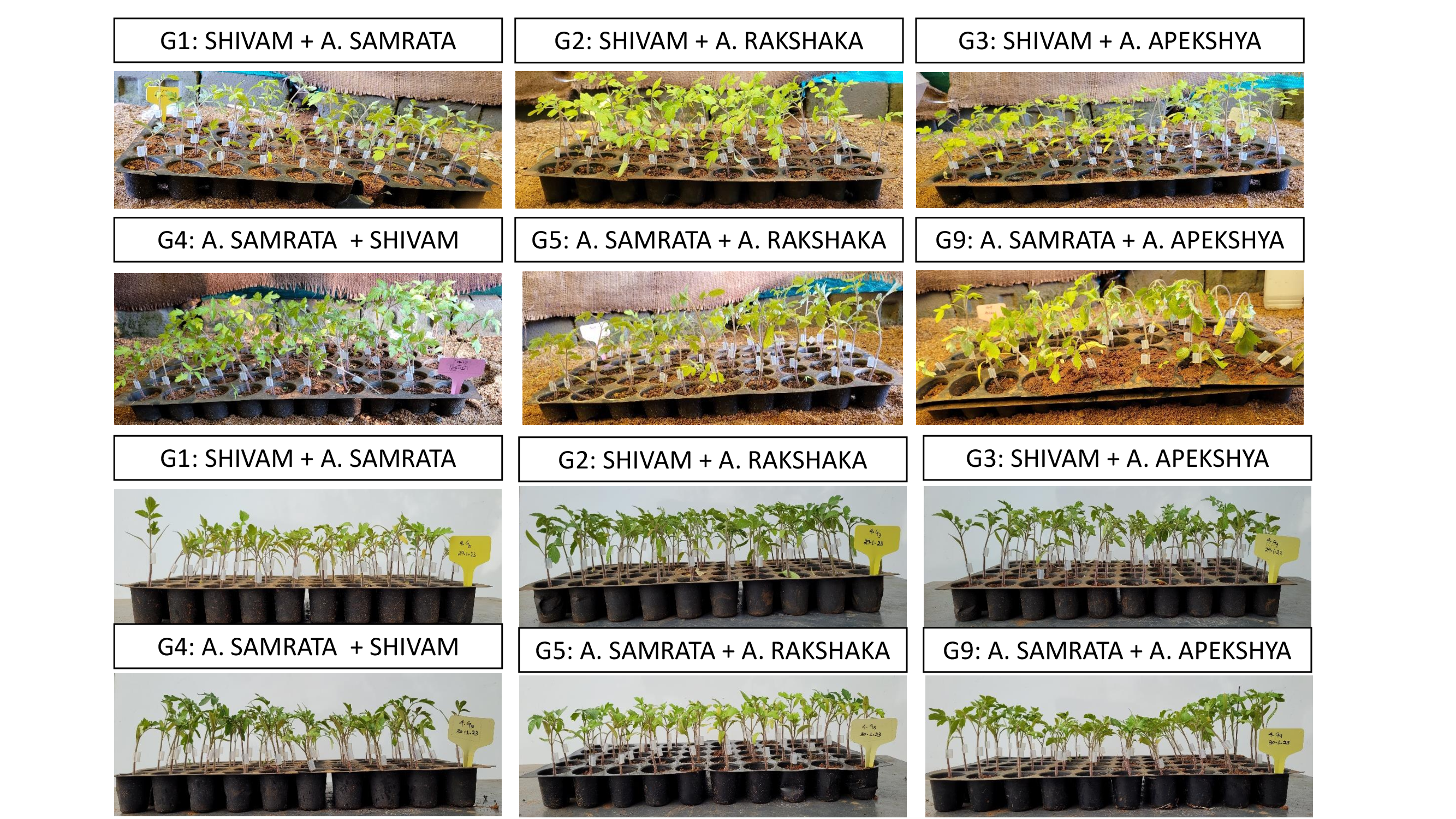

Supplement: Supplementary file 2 [file Image1.tiff]

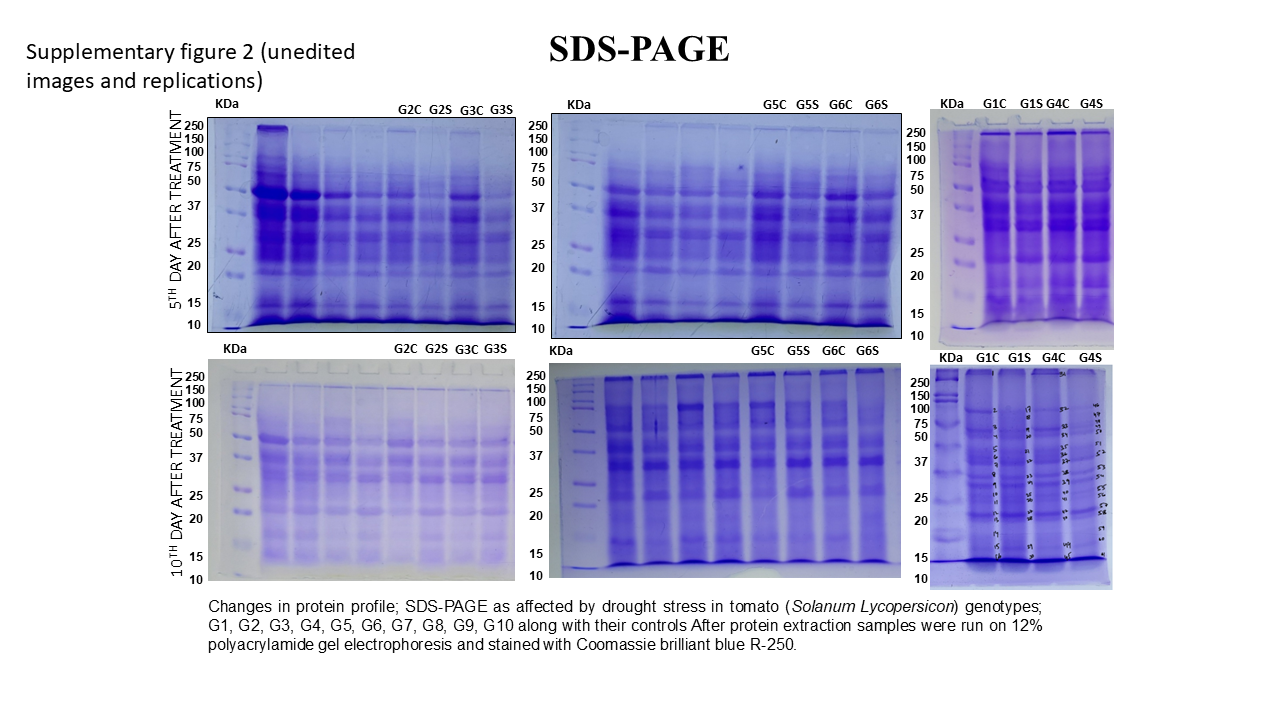

Supplement: Supplementary file 3 [file Image2.tif]
